# Diterpenes from the Brown Alga *Dictyota crenulata*

**DOI:** 10.3390/molecules13061253

**Published:** 2008-06-04

**Authors:** Joel Campos De-Paula, Ludmila Bomeny Bueno, Diana Negrão Cavalcanti, Yocie Yoneshigue-Valentin, Valéria Laneuville Teixeira

**Affiliations:** 1 Departamento de Biologia Vegetal, Instituto de Biologia Roberto Alcântara Gomes, Universidade do Estado do Rio de Janeiro, Rio de Janeiro, RJ, 20550-013, Brazil; E-mail: jc.paula@terra.com.br; 2 Programa de Pós-Graduação em Ciências Biológicas (Botânica), Museu Nacional, Universidade Federal do Rio de Janeiro, Rio de Janeiro, RJ, 20940-040, Brazil; 3 Programa de Pós-Graduação em Química Orgânica, Instituto de Química, Universidade Federal Fluminense, Niterói, RJ, Brazil; E-mail: ludmila.bomeny@globo.com; 4 Departamento de Biologia Marinha, Instituto de Biologia, Universidade Federal Fluminense, CP 100. 644, Niterói, RJ, 24001-970, Brazil; E-mail: dn.cavalcanti@uol.com.br; 5 Departamento de Botânica, Instituto de Biologia, Universidade Federal do Rio de Janeiro, Rio de Janeiro, RJ, Brazil; E-mail: yocie@biologia.ufrj.br

**Keywords:** *Dictyota crenulata*, Dictyotaceae, Phaeophyceae, diterpenes, chemotaxonomy

## Abstract

The crude extract of the Brazilian brown alga *Dictyota crenulata* was analyzed by NMR spectroscopy and HRGC-MS techniques. Seven diterpenes were identified: pachydictyol A, dictyodial, 4β-hydroxydictyodial A, 4β-acetoxydictyodial A, isopachy-dictyol A, dictyol C and dictyotadiol. Xeniane diterpenes have previously been found in *D. crenulata* from the Pacific Ocean. The results characterize *D. crenulata* as a species that provides prenylated guaiane (group I) and xeniane diterpenes (group III), thus making it a new source of potential antiviral products.

## Introduction

It has long been recognized that algae from the genus *Dictyota* Lamouroux present difficulties in the establishment of clear limits of separation between species. In previous studies, our group has indicated that the diterpenes from *Dictyota* species may have an important role as taxonomic markers [1,2,3,4,5,6,7]. In 2007, for example, a new species of the genus was established for the Brazilian coast based on morphological and chemical (diterpenes) characteristics [7].

Based on a revised biogenetic scheme proposed by Teixeira and Kelecom [1], the diterpenes have been distributed into three groups (I-III), depending on the structure of the products resulting from the the first formal cyclization of the geranyl-geraniol precursor. Thus, group I compounds result from a first cyclization of geranyl-geraniol between positions 1 and 10; group II compounds derive from a first cyclization of the precursor between positions 1 and 11, and group III compounds from either a first formal cyclization between positions 2 and 10 or by ring contraction of the prenylated germacrane.

The geographic distribution of *D. crenulata* is probably limited to the Atlantic and the western coast of Middle America [8]. De Clerck [8] noted that the basis on which Taylor separated *D. jamaicensis* from *D. crenulata* has never been clear, and several authors have questioned the status of *D. jamaicensis* [9,10,11,12]. Following the suggestion of these authors, *D. jamaicensis* is treated as a synonym of *D. crenulata*. *D. crenulata* is characterized by a multilayered medulla near the base of the thallus and spathulate apices.

**Figure 1:**
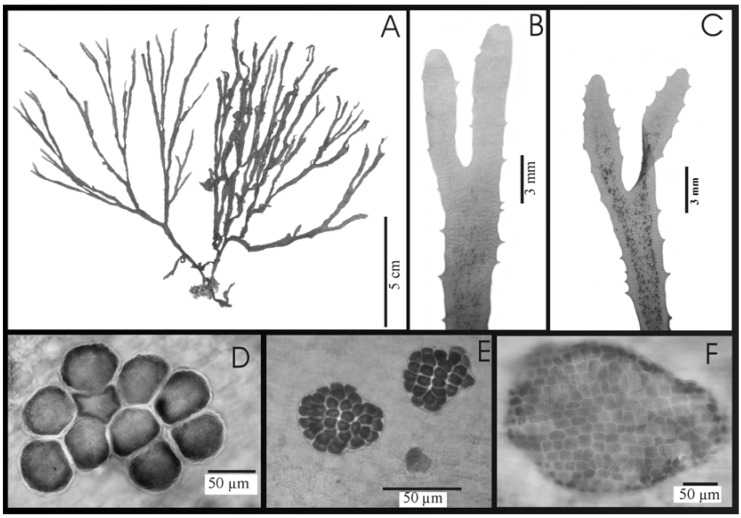
*D. crenulata* from Aracruz (ES), Brazil: A) External morphology; B) Detail of the apex with parallel ramification; C) Triangular to crenulate dentation with concentrated sporangia located in the center of the thallus; D) Superficial view of a sporangial sori; E) Superficial view of an oogonial sorus; F) Surface view of antheridial sorus.

*D. crenulata* has proven to be a rich source of diterpenes. Previous investigations of this alga collected from Hawaii yielded pachydictyol A, dictyodial, 4β-hydroxydictyodial A, 18, *O*-dihydro-4β-hydroxydictyodial A, 18,*O*-dihydro-4β-hydroxydictyodial A 18-acetate, and β-crenulal [13,14,15], whereas specimens from the Gulf of California yielded pachydictyol A and acetoxycrenulide [16], and specimens from Easter Island gave five prenylated guaianes and dictyocrenulol [17,18] (compounds **1**-**16**, Table 1).

**Figure 2:**
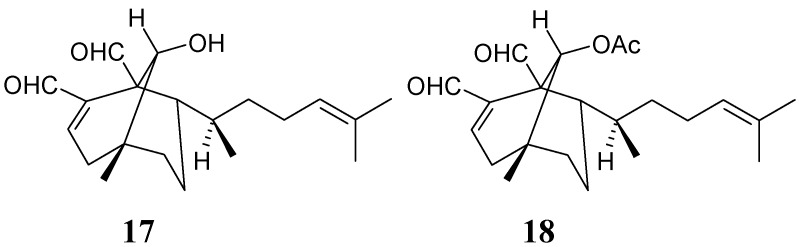


Four unsaturated diterpene dialdehydes: dictyodial (**10**) and 4β-hydroxydictyodial (**11**), previously isolated from *D. dichotoma* [19], and (6*R*)-6-hydroxydichotoma-3,14-diene-1,17-dial (**17**) and its acetate derivative (6*R*)-6-acetoxydichotoma-3,14-diene-1,17-dial (**18**), isolated from *D. menstrualis* [20] showed inhibitory effects against HIV-RT (reverse transcriptase enzyme) activity. The discovery and characterization of new anti-HIV agents and new sources with either novel structures or mechanism(s) of action and low toxicity to the host remain a priority [21]. The dichotomane diterpenes **17**-**18** (Figure 2) inhibited virus replication with EC_50_ values of 40 µM (12 µg/mL) and 70 µM (24 µg/mL), respectively. Furthermore, **17** and **18** reduced HIV-1-RT activity with IC_50_ values of 10 µM and 35 µM, respectively. Considering that neither **17** nor **18** affected either cell viability or proliferation, the synthesis of proviral DNA is a specific target for the anti-HIV-1 activity of the diterpenes [20]. The antiviral activities described for **17** and **18** are as effective as those described for the previously isolated diterpenes [19], dictyodial (**10**) and 4β-hydroxydictyodial (**11**), that showed EC_50_ values of 4.3 and 9.2 µg/mL, respectively. Thus, the presence of α,β-unsaturated dialdehydes might be a determinant for the expression of the inhibitory activity of these substances.

## Results and Discussion

Table 1 presents the diterpene composition of the *D. crenulata* collections from Hawaii [13,14,15], the Gulf of California, Mexico [16], from Easter Island [17,18] and from the Município de Aracruz, Espirito Santo State, Brazil. All the *D. crenulata* samples produced diterpenes from groups I (prenylated guaianes) and III (xenianes and its derivatives).

Table 2 presents the similarity matrix between the populations of *D. crenulata*. The highest indices were obtained for sample**s** that produced prenylated guaiane (group I) and xeniane diterpenes (group III): Gulf of California and Brazil (S = 0.55); Hawaii and Gulf of California (S = 0.44)**;** and Hawaii and Brazil (S = 0.57).

**Table 1:** Diterpenes from various collections of *D. crenulata* 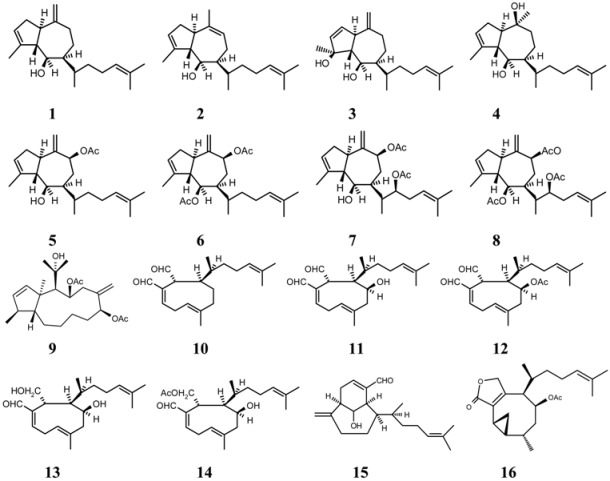

A) Group I :	Hawaii	Gulf of California	Easter Island	Brazil
	[13,14,15]	[16]	[17,18]	[this study]
pachydictyol A (**1**)	X	X	X	X
isopachydictyol A (**2**)				X
dictyotadiol(**3**)				X
dictyol C (**4**)				X
9-epidictyol B acetate (**5**)			X	
9-epidictyol B diacetate (**6**)			X	
dictyotriol A diacetate (**7**)			X	
dictyotriol A triacetate (**8**)			X	
dictyocrenulol (**9**)			X	
**B) Group III:**				
dictyodial A (**10**)	X	X		X
4β-hydroxydictyodial A (**11**)	X			X
4β-acetoxydictyodial A (**12**)				X
*18-O*-dihydro-4β-hydroxydictyodial A (**13**)	X			
18- *O*-dihydro-4β-hydroxydictyodial A 18 acetate (**14**)	X			
β-crenulal or sanadaol (**15**)	X			
acetoxycrenulide (**16**)		X		X

The lowest similarity indices (S = 0.14, 0.17 and 0.22) were obtained when the population from Easter Island was compared with the remaining populations. From the calculated similarity indices, it may be stated that the population from Easter Island is different and does not belong to the same species as the remaining populations. Therefore this sample needs to be reinvestigated for taxonomic purposes.

**Table 2:** Similarity matrix between populations of *D. crenulata*.

		1	2	3	4
Hawaii	1	1.00			
Gulf of California	2	0.44	1.00		
Easter Island	3	0.17	0.22	1.00	
Brazil	4	0.57	0.55	0.14	1.00

This is the first chemotaxonomic study of *D. crenulata*. With the exception of the Easter Island samples, the material collected from Pacific and Atlantic areas produced diterpenes from the chemical groups I and III. Specimens of this alga led to the isolation of six diterpenoids, five possessing a perhydroazulene skeleton.

4β-Acetoxydictyodial A (**12**) is the major product from Brazilian *D. crenulata* and acetylated 4β-hydroxydictyodial (**11**) from the Hawaiian population [15]. The results characterize *D. crenulata* as a species that furnishes prenylated guaiane (group I) and xeniane diterpenes (group III) and confirms the chemical similarity between Pacific and Atlantic populations and enlarges the geographical distribution of the xeniane diterpenes. The present data place *D. crenulata* chemically closer to others tropical western Atlantic species of *Dictyota*, such as *D. menstrualis* (Hoyt) Schnetter, Hörnig and Weber-Peukert and *D. ciliolata* Sonder *ex* Kützing. These two species differ chemically from *D. crenulata* by the majority production of pachydictyol A (**1**), dichotomane and cycloxeniane diterpenes [5] from *D. menstrualis*, and dictyol B acetate from *D. ciliolata* [23]. Since characterization of a species is the first step for its scientific use, the utilization of NMR spectroscopy and HRGC/MS techniques may be an excellent alternative for chemotaxonomic, seasonal variation, ecological and biogeographical studies.

### Structure Elucidation

The acetone extract of *D. crenulata* was analyzed by NMR spectroscopy. The NMR spectra of the crude extract and fractions revealed the presence of pachydictyol A (**1**), isopachydictyol A (**2**), dictyotadiol (**3**), dictyol C (**4**). dictyodial (**10**), 4β-hydroxydictyodial A (**11**), and 4β-acetoxydictyodial A (**12**). The latter was identified as the principal component in the mixture, along with dictyodial (1**0**) and 4β-hydroxydictyodial A (**11**). The identification of 4β-acetoxydictyodial A (**12**) was based on ^1^H- and ^13^C-NMR spectroscopic data comparison with literature values [22 and 15, respectively]. 4β-Hydroxydictyodial A (**11**) was identified in the same way [15]**.** We established that the OAc group is attached β to C-4, since the proton on C-4 (δ 5.30) shows small to nil couplings (0-4 Hz) to all of its vicinal neighbours. The presence of the alcohol **3 **in the mixture reinforces the β-orientation position of the OAc group.

Finally, after several attempts of purification of the xeniane diterpenes, substances **1-3** were transformed into crenulide diterpene products. The photoisomerization of xeniane into the crenulatane skeleton was previously reported [24]. The extract from *D. crenulata* was analyzed by HRGC-MS. This analysis revealed the presence of peaks in the mass spectra compatible with the fragmentation patterns of xeniane, **10**-**12**, and prenylated guaiane diterpenes **1**-**4**. The mass spectra of the diterpenes are summarized in Table 3.

**Table 3:** Principal peaks observed in the mass spectra (*m/z* in parenthesis) of diterpenes from *D. crenulata.*

Compound	Molecular formula	Rt *	Molecular Mass	Principal peaks
Pachydictyol A (**1**)	C_20_H_32_O	12.88	288	288 (M^+^), 270 (M^+^-H_2_O), 255 (M^+^-H_2_O-CH_3_), 227, 188, 177, 175, 159 (M^+^-H_2_O-side chain), 121, 107, 91, 69 (C_5_H_9_, base peak), 55, 41.
Isopachydictyol A (**2**)	C_20_H_32_O	13.14	288	288 (M^+^), 270 (M^+^-H_2_O), 255 (M^+^-H_2_O-CH_3_), 227, 188, 173, 159 (M^+^-H_2_O-side chain, base peak), 145, 119, 107, 91, 69 (C_5_H_9_), 55, 41.
Dictyotadiol (**3**)	C_20_H_32_O_2_	16.40	304	304 (M^+^), 286 (M^+^-H_2_O), 271 (M^+^-H_2_O-CH_3_), 243, 219, 201, 187, 175, 159 (M^+^-2H_2_O-side chain), 133, 121, 109, 95, 82 (base peak), 69 (C_5_H_9_), 55.
Dictyol C (**4**)	C_20_H_34_O_2_	15.82	306	306 (M^+^), 288 (M^+^-H_2_O), 270 (M^+^-2H_2_O), 255 (M^+^-2H_2_O-CH_3_), 245, 203, 185, 177, 173, 159 (M^+^-2H_2_O-side chain), 145, 121, 119, 81, 69 (C_5_H_9_,base peak), 55, 41.
Dictyodial (**10**)	C_20_H_30_O_2_	18.49	302	302 (M^+^), 284 (M^+^-H_2_O), 269 (M^+^-H_2_O-CH_3_), 187, 173, 109, 107, 93, 69 (C_5_H_9_, base peak), 55.
4β-Hydroxydictyodial A (**11**)	C_20_H_30_O_3_	20.10	318	300 (M^+ ^- H_2_O), 201, 189, 177, 176, 165, 161, 147, 135, 109, 95, 91, 82, 69 (C_5_H_9_, base peak), 67, 55.
4β-Acetoxydictyodial A (**12**)	C_22_H_32_O_4_	20.98	360	300 (M^+^-AcOH), 282 (M^+^-AcOH-H_2_O), 201, 189, 177, 176, 161, 147, 135, 109, 95, 93, 91, 69 (C_5_H_9_, base peak), 55.
Acetoxycrenulide (**16**)		24.27		316, 298, 281, 253, 235, 217, 190, 109 (base peak), 91, 69 (C_5_H_9_), 55.

* Retention time in minutes

## Experimental

### General

All solvents were HPLC grade. Analytical thin-layer chromatography (TLC) separations were carried out on Merck silica gel 60 F-254 (0.2 mm) precoated aluminum plates. Once developed, plates were visualized by spraying with 2% ceric sulphate in sulfuric acid, followed by gentle heating. Silica gel 60 (Merck, 70-230 and 230-400 mesh) was used for column chromatography. NMR spectra were recorded in CDCl_3 _(100% Aldrich) on a Varian Unity Plus 300 spectrometer using TMS as internal standard. The preliminary gas chromatographic analysis (HRGC) was carried out on an HP 5890 CG machine equipped with an SE-54 glass capillary column (15 m x 0.25 mm; film thickness 0.25 µm) using a FID detector. The extract was analyzed by HRGC-MS on a HP 6890 series GC system, coupled to a HP 5973 mass selective detector in the electron impact mode (70 eV) equipped with an HP-1 MS capillary column (30 m x 0.25 mm; film thickness 0.25 µm). Injector and detector temperatures were set at 270 ^o^C and 290 ^o^C, respectively. The temperature program was kept at 160 ^o^C, then programmed to 260 ^o^C at a rate of 4^o^ C/min and finally raised at a rate of 15 ^o^C/min to 290 ^o^C for 15 min. Hydrogen was the carrier gas at a flow rate of 1 mL/min. Diluted samples were injected manually in the split mode (1:10 or 1:20). Data were obtained from Frd area percent values. The chemical components were identified based on mass spectral comparison with those of standards and/or literature data*,* by co-injection in HRGC of these samples and from the Wiley 275 library data of the HRGC/MS system.

### Sample Collection

Specimens of *D. crenulata* were collected at Praia dos Padres, Portocel, Município de Aracruz, Espirito Santo State (lat. 19^o^49’24”S, long. 40^o^03’00”W), Brazil during June 2003. The seaweeds were washed with local sea water, separated from sediments, epiphytes and other associated organisms. The algae were collected by Dra. Cristina Nassar (Universidade Federal do Rio de Janeiro) and identified by Dra. Cristina Nassar and Dr. Joel Campos de Paula. Voucher specimens are deposited at the herbarium of the Universidade do Estado do Rio de Janeiro (HRJ 10326).

### Extraction and Isolation

Air-dried *D. crenulata* (100 g, dry weight) was extracted with acetone (100%) at room temperature. Evaporation of the solvent under reduced pressure yielded the crude extract (1.2 g) as a brownish residue. The partial crude extract (951 mg) was subjected to silica gel column chromatography (3 x 40 cm) eluting with *n*-hexane/acetone (9:1) to give a total of 37 fractions. The fractions yielded three compounds, the major being identified as 4β-acetoxydictyodial (**12**) and dictyodial (**10**) and 4β-hydroxydictyodial (**11**) as minor components.

### Similarity Matrix

The degree of affinity between the algal populations of each area of collection was deduced from the similarity index (S) following Sørensen [25]. This index was calculated using the diterpene skeletons that are common to both considered populations, and using for each skeleton the lowest number of common diterpenes. This index was preferred over others since it attributes a higher weight to positive characters (here diterpene skeletons) than to negative ones.
